# 4SM: A Novel Self-Calibrated Algebraic Ratio Method for Satellite-Derived Bathymetry and Water Column Correction

**DOI:** 10.3390/s17071682

**Published:** 2017-07-21

**Authors:** Yann G. Morel, Fabio Favoretto

**Affiliations:** 1 Retired, BP 2862 98703 Punaauia, French Polynesia; 2 Posgrado en Ciencias Marinas y Costeras (CIMACO) Universidad Autonoma de Baja California Sur, La Paz Baja California Sur 23080, Mexico; favoretto.fabio@gmail.com

**Keywords:** remote sensing, attenuation coefficient, ratio method, soil line, bottom reflectance, shallow substrate, satellite derived bathymetry, water column correction, bottom typing

## Abstract

All empirical water column correction methods have consistently been reported to require existing depth sounding data for the purpose of calibrating a simple depth retrieval model; they yield poor results over very bright or very dark bottoms. In contrast, we set out to (i) use only the relative radiance data in the image along with published data, and several new assumptions; (ii) in order to specify and operate the simplified radiative transfer equation (RTE); (iii) for the purpose of retrieving both the satellite derived bathymetry (SDB) and the water column corrected spectral reflectance over shallow seabeds. Sea truth regressions show that SDB depths retrieved by the method only need tide correction. Therefore it shall be demonstrated that, under such new assumptions, there is no need for (i) formal atmospheric correction; (ii) conversion of relative radiance into calibrated reflectance; or (iii) existing depth sounding data, to specify the simplified RTE and produce both SDB and spectral water column corrected radiance ready for bottom typing. Moreover, the use of the panchromatic band for that purpose is introduced. Altogether, we named this process the Self-Calibrated Supervised Spectral Shallow-sea Modeler (4SM). This approach requires a trained practitioner, though, to produce its results within hours of downloading the raw image. The ideal raw image should be a “near-nadir” view, exhibit homogeneous atmosphere and water column, include some coverage of optically deep waters and bare land, and lend itself to quality removal of haze, atmospheric adjacency effect, and sun/sky glint.

## 1. Introduction

Since the advent of LANDSAT MSS, most projects of passive remote sensing of SDB required the use of at least a few existing bottom depth points to derive empirical linear regressions between water depths and measured spectral radiance or reflectance at the base of the atmosphere. They use a simplistic statistical equation, assume homogeneous optical properties of the atmosphere and water column, and do not consider the optical properties of the water column.

Most empirical approaches only yield SDB [1,2], but they are fairly simple and fast to operate, and therefore widely used. Although they have been recently reported to be useful to the hydrographic community [3], and feature in the International Hydrographic Organisation’s cookbook [4] and in a commercial package [5], they have long been known to yield biased results over highly reflecting seabeds at very shallow depths, and also over poorly reflective seabeds [6,7]: if at all possible, they need to be improved [8,9]. In addition, a simple operational work flow must be offered in order to process the recent abundance of freely available quality image data like LANDSAT-8.

In contrast, since the turn of the century, several analytical methods use a general optical equation dependent on intrinsic optical properties of sea water, illumination and viewing geometry, atmospheric and glint effects, etc. [10]. They require a look-up table (LUT) of spectral reflectance for all possible bottom substrates over the whole shallow depth range, for a wide range of those many factors. Then for each shallow pixel, the correct depth is chosen through a computer-intensive spectral matching optimization technique, which also yields the water column corrected spectral bottom reflectance and the spatial variations of the optical properties of the water column. This approach has recently shown promise in the hands of several teams of advanced R&D scientists using a very complex work flow and powerful computing [11], and has even been implemented in a commercial software package [12].

In this paper, a well-researched and accepted simplified RTE is summarized, and several new assumptions are introduced to operate it in a band combination approach. Then, the proposed work flow to derive both the SDB and the water column corrected spectral reflectance of the shallow bottom ready for bottom typing is detailed and illustrated. This work flow does not require the conversion of the relative radiance data into calibrated reflectance, and, most importantly, does not require existing depth sounding data to calibrate the RTE. This was first presented in 1998 by Morel and Lindell [13], and is used by Favoretto et al. [14].

The following paper draws on the 23-year study, in quasi-isolation, of hundreds of multispectral and hyperspectral images, airborne and spaceborne, worldwide: aerial photography, LANDSAT, SPOT, QUICKBIRD, WORLDVIEW-2, Compact Airborne Spectrographic Imager (CASI), HYPERION, even Hyperspectral Imager for the Coastal Ocean (HICO). As presented at [15], this study reflects the experience gained at sea level, but also at high altitude (melt water ponds in Greenland, briny shallow lakes in Altiplano), under clear skies, but also under overcast skies in the Middle East. It includes several time series of LANDSAT and WORLDVIEW-2 images, and covers from very high (1 m GSD) to very coarse spatial resolution (100 m GSD).

For a recent and comprehensive introduction to the basics of water column correction, and to no fewer than 18 methodological approaches, sorted into band combination, model-based algebraic, optimization/matching, and multi-temporal analysis categories, please refer to a review by Zoffoli et al. [16]. These authors view the simplified RTE approach as an algebraic model-based algorithm, and conclude: “*More complex algebraic algorithms have been developed to estimate the reflectance in shallow environments that require more field data. They are the only methods capable of estimating bottom reflectance. For this reason, improvements and validations of this group of algorithms should be encouraged.*” For more reviews, please also see Dekker et al. [11], and Carmody [8].

The purpose of this article is to show that, in the 4SM approach, the calibration of the simplified RTE does not require the use of any existing depth sounding information to yield an estimate of both the water depth and the water column corrected spectral bottom reflectance.

## 2. The Simplified RTE

Preamble: as shall be exposed in Section 2.2, inverting the model (3) in the model-based algebraic 4SM approach amounts to simplistic spectral matching. Therefore it does not require L radiance terms to be converted into physical units of reflectance R: this is the advantage and privilege of a “ratio method”, which is only concerned with ratios among numbers. Therefore, in all that follows in this article, the terms reflectance or radiance or digital number (DN) are used interchangeably, meaning the relative intensity of the signal captured by the sensor.

### 2.1. An Operational Model

From albedo *A* and reflectance *R*, to radiance *L*, or to digital numbers, the reader is expected to have a fair understanding of the definitions and concepts in ocean color remote sensing. The spectral reflectance of shallow bottoms *R* or *L*, as measured at the base of the atmosphere (BOA), has suffered the diffuse attenuation of the solar light flux on its way down from sea-surface to shallow bottom, then on its way up from shallow bottom to sea-surface (1,2). This is described by the following simplified RTE, for Case-1 waters where the diffusion by mineral particles may be considered very low. This RTE has been established and verified using irradiance measurements in water. Here it must be remembered that measuring the irradiance of the light flux, whether downward or upward, accounts for the fate of all photons in a hemisphere. In contrast, remote sensing devices collect photons through an extremely narrow field of view; this shall be discussed in Section 6.3.

The simplified RTE was formulated by Philpot [17], then by Maritorena et al. [18]:

(1) $$\mathrm{BOA}:\;R=Rw+\frac{A-Rw}{e^{2KZ}}.$$

It is rewritten for BOA radiances *L* at the base of the atmosphere:

(2) $$\mathrm{BOA}:\;L=Lw+\frac{LB-Lw}{e^{2KZ}}\;.$$

It is now inverted to yield the BOA water column corrected reflectance *LB*:

(3) $$\mathrm{BOA}:\;LB=Lw+\left(L-Lw\right)e^{2KZ}.$$

Add the atmospheric path radiance *La* to all *L* terms in (2):

(4) $$\mathrm{TOA}:\;L+La=Lw+La+\frac{LB+La-Lw-La}{e^{2KZ}}.$$

So the simplified RTE at the sensor becomes:

(5) $$\mathrm{TOA}:\;Ls=Lsw+\frac{LsB-Lsw}{e^{2KZ}}.$$

Estimating the atmospheric path radiance after removal of any glint:

(6) La=Lsw−Lw.

Estimating the BOA measured radiance after removal of any glint:

(7) L=Ls−La.

Linearizing the bottom contrast:

(8) Linearization: X=log(Ls−Lsw).

In these equations:

- BOA stands for “at the base of the atmosphere”, commonly dubbed “water-leaving”.
- TOA stands for “at the top of the atmosphere” or “at the sensor”.
- *A* is the albedo of the shallow bottom, dimensionless.
- *R* is the reflectance of the shallow bottom measured at sea-surface, dimensionless.
- *Z* is the height of the water column, or depth of the shallow substrate, in units of m.
- 2*K* is an operational two-ways attenuation term in units of m^−1^, the sum of a down-welling term and an up-welling term: 2*K* = *K*down + *K*up.
- 2*K*·*Z* is the optical depth, or two-ways optical path length, dimensionless.
- *LB* is the spectral BOA radiance of the bottom substrate at null depth.
- *LsB* is the spectral TOA radiance of the bottom substrate at null depth.
- *LsM* is the maximum TOA value for *LsB*, for the maximum reflectance of a shallow bottom at null depth on site.
- *Lw* is the spectral BOA radiance of the sea where the bottom is optically deep, or backscatter.
- *Lsw* is the spectral TOA radiance of the sea where the bottom is optically deep, also called water volume reflectance.
- *L* is the spectral BOA radiance of the bottom substrate at depth *Z*.
- *Ls* is the spectral TOA radiance of the bottom substrate at depth *Z*.
- *La* is the spectral radiance of the atmospheric path, measured from satellite altitude, also called path radiance.
- *s* in *LsB*, *LsM*, *Lsw*, and *Ls* stands for “at the sensor” or TOA.

Please note:

- Wavelength dependency subscripts of all *L* and *K* terms are omitted for brevity**.**
- Operational *K*: in the following, *K* and 2*K* are used interchangeably, for brevity. Replacing *K*down + *K*up by 2*K* is a very practical simplification. This is discussed extensively by Maritorena et al. [18]; these authors conclude: “*The approximate formulae can be safely adopted in operation when interpreting or predicting the reflectance of shallow waters, in particular if Rw and Kd have been estimated from remotely sensed data.*”
- De-glinting: Any contribution to the measured radiance by the haze, the atmospheric adjacency effect, or the sun/sky glint must first be removed, as the simplified RTE applies at sea level (BOA).
- Most importantly, it is assumed that the atmosphere and the shallow water exhibit homogeneous optical properties over the whole scene.

**Figure 1:**
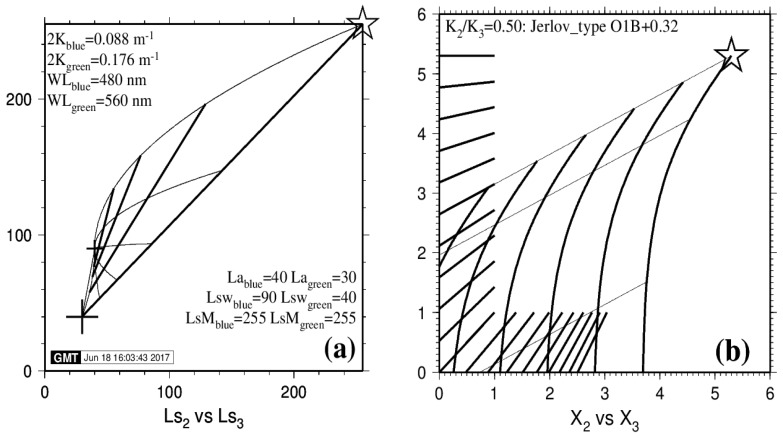
(**a**) Operating the RTE (Equation (5)) at the top of the atmosphere: A plot of *Ls*_blue_ versus *Ls*_green_ in relative units. (**b**) Operating the RTE (Equation (8)) for linearized data: A plot of *X*_blue_ versus *X*_green_ in logarithmic values.

In the 4SM approach, it is important to understand the inner workings of the simplified RTE. For that purpose, the graphic illustrations of Figure 1a,b help to take control of how things work. This kind of graph is an exclusive and valuable bonus to the 4SM approach. They have been prepared using our 4SM code (see Section 4.1), but may as well be prepared using a spreadsheet. In these graphs, the Soil Line and the Brightest Pixels Line (BPL) are introduced: these terms shall be defined in Section 3.1 and Section 3.2. Band 2 is the blue band; Band 3 is the green band.

In Figure 1a, values are in relative radiances scaled to floating point in the range 0–255. The values for *La* (big cross) and *Lsw* (small cross) have been set in order to convey a clear message in Figure 1a, rather than to be physically realistic. Star denotes *LsM*; small cross denotes *Lsw*; big cross denotes path radiance *La*. Six thin curved lines represent the exponential decay for six bottom types (*LM*_blue_: 215, 107.5, 53.8, 26.9, 13.4, 0); the first is the BPL; two of them are darker than the deep water reflectance *Lsw* (small cross): They exhibit a negative bottom contrast where *LB* < *Lw*; and the last one represents a black body. Four thick straight lines represent four isobath lines: 0 (the Soil Line), 5, 10 and 15 m.

In Figure 1b, radiance values are linearized by application of Equation (8). Three thin straight lines represent three bottom types: They exhibit a positive bottom contrast where *Ls* > *Lsw*; their slope is the ratio *K*_2_/*K*_3_, or *K*_blue_/*K*_green_. Six thick curved lines represent six isobath lines: 0 (the Soil Line), 5, 10, 15, 20 and 25 m. The Soil Line is a curved line: This is because *Lw*_blue_ is distinctly stronger than *Lw*_green_. However, in the case of a Red versus NIR pair of bands, the Soil Line would be a straight line, because *Lw*_red_ and *Lw*_NIR_ are both negligible.

It should be noted that computing a multiple linear regression using existing depth data in empirical methods [1,2] amounts to placing a straight line at null depth in Figure 1b, and use it as a radiometric reference model at null depth, somewhere over and in place of the curved Soil Line, with a slope and an intercept, and with all isobath lines being straight and parallel to that reference line. As signaled in the introduction, this results in various bias in retrieved depth, particularly in substantial depth underestimation over dark bottom substrates.

All parameters of the simplified RTE are now written down and well understood, so that this model may now be inverted in order to retrieve the depth and spectral reflectance of the shallow bottom.

### 2.2. Inverting the Model

Attenuation means that the bottom reflected signal L in Equation (2) decreases as the height of the water column increases. Inverting the model means that, by increasing *Z* in Equation (3), the water column corrected reflectance *LB* gets restored back to where it belongs on the Soil Line. In practice, this is done as follows: Let *LB_j_* denote the water column corrected radiance for waveband *j*, which suffered the strongest attenuation (like the Red waveband); and let *Lb_i_* denote the average of all *LB* terms for wavebands *i* which suffered weaker attenuation (like all Blue-Green wavebands).

In the 4SM approach, inverting the model is done by increasing *Z* in Equation (3), until the ratio *LB_i_*/*LB_j_* matches that of an operational radiometric model observed over bare land areas at sea level for the same band combination. We call this model the Soil Line, and it shall be introduced in Section 3.1. As a practical measure, if all BOA radiance terms are normalized, then the slope of the Soil Line is conveniently scaled to 1; so that the inversion proceeds until the ratio *LB_i_*/*LB_j_* equals 1. Section 5.3 shall show that variants may be applied to this rule.

Several solutions may be operated to solve for spectral *LB* and depth *Z*, depending on the bottom contrast *Ls*-*Lsw*. In the case of a LANDSAT-8 image, and using only bands that exhibit significant bottom detection, a pixel may be modeled using:

- Either the NIR solution:
  (9) $$\frac{LB_{1}+LB_{2}+LB_{3}+LB_{4}}{4LB_{5}},$$
- or the Red solution:
  (10) $$\frac{LB_{1}+LB_{2}+LB_{3}}{3LB_{4}},$$
- or the Green solution:
  (11) $$\frac{LB_{1}+LB_{2}}{2LB_{3}},$$
- or the Pan solution:
  (12) $$\frac{LB_{1}+LB_{2}+LB_{3}}{3LB_{\mathrm{PAN}}}.$$

Please note that, if the Pan solution is used, it shall cover the whole depth range seamlessly. This all-important and beneficial aspect shall be discussed in Section 6.6.

## 3. Three New Simplifying Assumptions in 4SM

Inverting the model requires the following four things, which can be estimated from the image itself:

- An operational spectral radiometric model of bare land areas at sea level, where depth *Z* is null;
- spectral *Lsw*, spectral *Lw*, spectral *La*, and spectral 2*K* in units of m^−1^.

For this to be done without the use of any existing depth sounding, the 4SM approach introduces three new simplifying assumptions, namely the Soil Line assumption, the Brightest Pixels Line assumption, and the Jerlov assumption.

### 3.1. The Soil Line Assumption: Estimating Spectral LsM, Lsw, Lw, and La

In the 4SM approach, the claim that there is “no need for atmospheric correction” rests on the investigation of the image to specify the spectral atmospheric path radiance *La* and the spectral water volume reflectance *Lw*. This in turn rests on the specification of the Soil Line, which shall now be examined.

Bare land pixels at sea level in the image are sampled and analyzed in order to specify the Soil Line, i.e., an operational BOA radiometric model at null depth. The Soil Line is estimated as follows:

- Let *LB*max be the BOA reflectance of brightest bottom substrate at null depth, like over fine clean coral or quartz beach sand;
- let *LB* = 0 be the null BOA reflectance, like that of a black body at null depth.

The bare land pixels in the image at sea level, from brightest to darkest, are assumed to provide a suitable operational spectral model of bare shallow bottoms at null depth. This is intended to represent all shades of gray in the screen display of an enhanced color composite of the image, from black (*LB* = 0) to white (*LB* = *LB*max), and would plot along a straight line in a diagonal position in a bi-dimensional histogram of normalized BOA radiances.

In the case of a Blue versus Green bi-dimensional histogram, a greenish shallow bottom pixel at null depth, like a green vegetated pixel, would plot on the right hand side of the Soil Line in Figure 1a. Of course, this shall be the cause of a possibly severe underestimation of the retrieved depth unless something is attempted to alleviate this depth residual; and vice versa for a blueish shallow bottom pixel. This aspect shall be discussed in Section 5.3.

Now that the Soil Line is specified, how we specify the atmospheric path radiance *La*, and the water volume reflectance *Lw*, are specified. Estimating the TOA deep water radiance *Lsw* from the image requires that the scene under investigation exhibits an area that is optically deep in all image bands. It also requires preliminary de-glinting, i.e., the removal of the contribution of the reflection of sky/sun light at sea-surface, and also of the adjacency effect.

Equation (2) is for BOA radiances. It features *Lw*, the BOA spectral radiance of the sea where the bottom is optically deep. This term is assumed to be ~null over the Red-NIR range; but it is certainly not negligible over the Blue-Green range, as it determines the color of the sea. An operational value of *Lw* over the Blue-Green range is estimated, using some common sense, through visual inspection of bi-dimensional histograms that exhibit the bare land pixels.

Then, remembering that *Lsw* is the TOA color of the optically deep sea after removal of any glint contribution, and that *Lw* cannot be negative, the atmospheric path radiance may be estimated as *La* = *Lsw* − *Lw* (Equation (6)). This is discussed further in Section 6.2.

### 3.2. The Brightest Pixels Line (BPL) Assumption: Estimating the Ratio K_blue_/K_green_

In order to estimate the ratio *K_i_*/*K_j_* of diffuse attenuation coefficients for any pair of bands *i* and *j* with *K_i_* < *K_j_*, the BPL is introduced as a radiometric model of the brightest shallow bottom substrate *LsM* in the image over the full depth range. The BPL represents the exponential decrease of the TOA radiance *LsM* as the bottom depth *Z* increases: This decrease is caused by the two-ways diffuse attenuation of the light flux through the water column.

Lyzenga observed in 1978 [1], that all pixels representing the same bottom substrate, e.g., homogeneous sandy bottoms at various depths, display along a straight line in a bi-dimensional histogram of linearized data *X* = log(*Ls* − *Lsw*) for bands *i* and *j*. The slope of this straight line is the ratio *K_i_*/*K_j_* of the diffuse attenuation coefficients *K_i_* and *K_j_*. However, Maritorena [19] was not able to secure a faithful value of the ratio *K*_green_/*K*_red_ over coral reef sands from a SPOT image of the island of Moorea, French Polynesia, using a manual selection of homogeneous sandy bottom regions of interest at various depths, as prescribed by Lyzenga. He then had to use a SPOT image of the island of Bora Bora, French Polynesia, where “*the lagoon is wide … [and] supposed to have large homogeneous bottom zones.*”

In contrast, it is observed that the brightest pixels in such a selection most often display as a straight line; this represents a significant variant of Lyzenga’s method where the ratio *K_i_*/*K_j_* is established through linear regression of all selected pixels. Therefore, in the 4SM approach, the ratio *K_i_*/*K_j_* for all pairs of wavebands may be specified using an algorithm that automatically selects the brightest pixels *Ls_i_* for all pairs of pixels *Ls_i_* and *Ls_j_* in the shallow areas of the image. In other words, Lyzenga’s observation applies to the brightest bottoms, which represent a physical limit of maximum brightness. As it turns out, this is mostly observed at isolated pixels rather than over extended supposedly homogeneous regions of interest, and may be verified by inspection of a screen display of the water column corrected reflectance. This allowed us to process the SPOT 1 image of Moorea in 1995 [20].

It should be noted that all ratios form a consistent suite, where *K_i_*/*K_k_* = (*K_i_*/*K_j_*)/(*K_k_*/*K_j_*) for all pairs of *K_i_*, *K_j_* or *K_k_*. Then, for the model to be inverted, spectral *K* for all visible bands may be derived by application of a seed value in this suite, in units of m^−1^: This seed value may be any of *K_i_*, *K_j_* or *K_k_*.

### 3.3. The Jerlov Assumption: To Get a Seed Value, and Specify Spectral K in m^−1^ over the Visible Range

Jerlov [21] (Page 70, Table XVII) proposed a classification of the diffuse attenuation properties for downwelling irradiance for marine waters worldwide under the following conditions: Clear sky, sun high in the sky, very low content in suspended mineral particles. He pointed out that “*the spirit of this classification is that the irradiance attenuation coefficients Kd for any wavelength can be expressed as a linear function of a reference wavelength.*”

Kirk [22] further commented: “*The reflectance spectra of oceanic waters vary in a roughly systematic way. A family of curves, of progressively changing shape, determined mainly by the phytoplankton concentration, is observed. Thus, for any given oceanic water, specification of the ratio of radiances or radiance reflectances at any two wavelengths, should in effect specify the whole radiance reflectance curve, and therefore the optical character of the water.*”

Using the ratio *K*_blue_/*K*_green_ observed in the image, experience showed over the years that spectral *K* for all visible bands (400 to 700 nm) may be interpolated from Jerlov’s family of curves. As it turns out,

- Blue-Green range: By application of interpolated spectral *K*, one obtains a tight fit of the slope *K_i_*/*K_j_* with the display of the brightest shallow pixels in a linearized bi-dimensional histogram for the pair of bands *i* and *j* in the Blue-Green range of the solar spectrum;
- ultraBlue range and Orange-Red range: Spectral *K* must be distinctly reduced manually in these two ranges. This only became clear recently, by use of closely spaced quality hyperspectral bands of the HYPERION and then HICO sensors [23];
- self-calibration: In practice, all sea truth regressions allow one to conclude that depths retrieved in meters under such conditions only need tide correction. This shall be presented in Section 5.

In summary, the simplified RTE describes the exponential decay of the reflectance of a shallow bottom as its depth increases. In order to solve for both *LB* and *Z* in Equation (3), the following new assumptions were introduced:

- In a bi-dimensional histogram of natural data, the Soil Line represents a spectral model of water column corrected reflectance *LB*.
- An optically deep area in the image is de-glinted and sampled in order to determine spectral deep water reflectance *Lsw*.
- Then an operational, and suitable, value of the spectral water volume reflectance *Lw*, and also of the spectral path radiance *La*, is derived by inspection of vegetated pixels in such bi-dimensional histogram for the pairs Blue versus Red and Green versus Red, and by using some common sense knowledge.
- In a linearized bi-dimensional histogram for the pair of bands *i* and *j*, with *K_i_* < *K_j_*, the ratio *K_i_*/*K_j_* is measured as the slope of the display of the brightest linearized pixels in band *i* for the whole range of linearized values in band *j*.
- Then this ratio for the Blue versus Green pair of bands is used to interpolate *K*_blue_ and *K*_green_ using Jerlov’s data. It follows that, in the end, *K* is specified for all other visible bands.

Now that all aspects of the 4SM approach have been presented (its equations, and the new assumptions), we proceed to explain how to work out all parameters that are needed to specify the simplified RTE (optical calibration) for the specific image to be processed.

## 4. Materials and Methods

**Figure 2:**
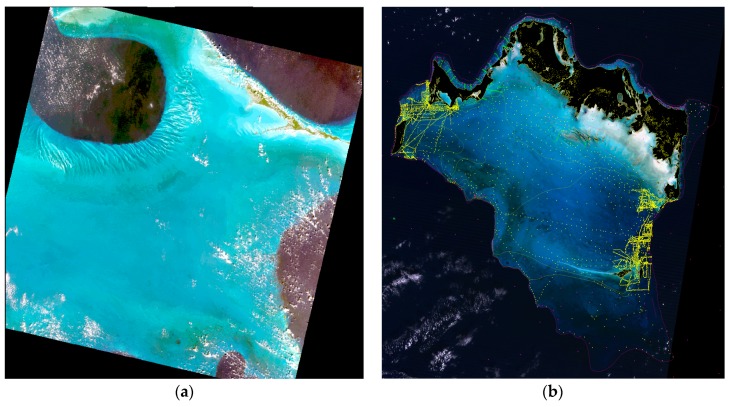
(**a**) LANDSAT-8 over Lee Stocking island, Bahamas (Tongue of the Ocean). (**b**) LANDSAT-8 over Caicos Bank, Bahamas. This view shows the location of three depth datasets combined by Morgan and Harris [24] to prepare their DTM, as detailed in Section 5: (i) yellow points show actual depth sounding points; (ii) yellow lines show isobaths lines extracted from the nautical chart; (iii) purple points show depths retrieved from Landsat TM imagery.

First, in Section 4.2, we shall illustrate the process of the optical calibration of the RTE without any use of existing depth soundings. For this, we use a 180 km wide LANDSAT-8 image over Oceanic waters at Lee Stocking Island, Bahamas, dated 29 January 2014 (Figure 2a), downloaded from USGS Earth Explorer.

Then, in Section 5.5, we shall illustrate a sea truth regression in order to verify that the 4SM approach does not need any existing depth sounding. A sea truth digital terrain model (DTM, 120 km wide) at 30 m ground sampling distance (GSD) of Caicos Bank, Bahamas, was purchased over the Internet from Harris-Ellis_Knowledge_Transfer project [24]; it may be inspected at [25]. It shall be used in Figure 4 and Figure 5, to evaluate the sea truth on depths retrieved using a LANDSAT-8 image dated 8 November 2014, downloaded from USGS.

### 4.1. The 4SM Code and Its Performance

We use the following open source resources:

- OpenEV as a lightweight image viewer, to export the raw data (usually a TIF file) into a PCIDSK formatted file, and to create shapefiles;
- system calls by 4SM code to GMT, the Generic mapping Tool to prepare PostScript files, and to Ghostview or Evince to display PostScript files on screen.

Apart from the above open-source resources, we only used our own 4SM code written in C on an x86_64 full HD notebook under Linux. This code was developed over the last 20 years as an ergonomic one-stop “do it all” proprietary code, under the control of one single command line bash script and a close supervision by the practitioner, with the following work flow that covers all aspects of the work:

- Import (and re-sample as appropriate) raw data into a working FILE interleaved PCIDSK formatted database;
- run through a series of preparations that include UTM georeferencing of that database, reading and using shapefiles, recoding of rasters and production of masks, optical calibration of the RTE, etc.;
- then, once satisfied with these image preparations, read up to 26 raw spectral bands, line after line,
- and proceed “on the fly”, for each line, to de-glinting, pan-sharpening if required, smart-smoothing if required,
- then apply the inverted simplified RTE to each shallow pixel,
- to produce a raster of SDB, several rasters of de-glinted bands, and several rasters of water column corrected bottom reflectance ready for bottom typing.

As regards performance on a 64-bit 2.4 GHz Intel I7 Core laptop, this code processes the 16.7 million pixels of the Caicos Bank LANDSAT-8 seven bands study case of Figure 2b in 3.1 min (that is 89,600 pixels per second), or a 17-band 36 million pixels 1 m GSD CASI image of Heron Island, Great Barrier Reef, in 54 min.

**Figure A1:**
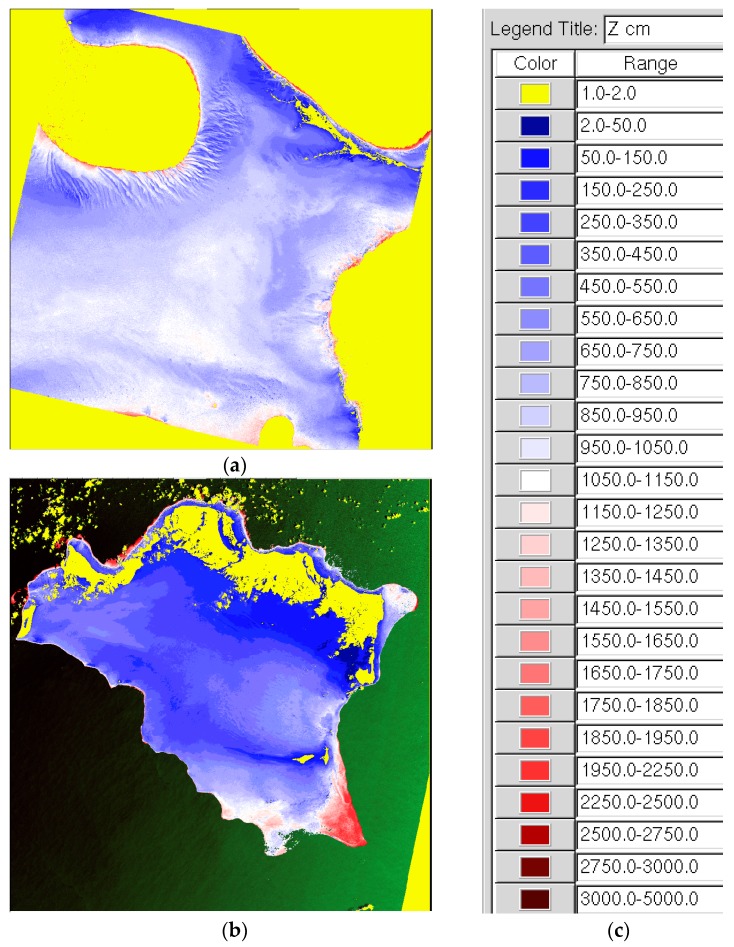
Satellite-derived bathymetry maps. (**a**) Combined depth for a time series of 14 Landsat-8 scenes over Lee Stocking Island, Bahamas. (**b**) SDB for a Landsat 8 image 19 April 2016 over Caicos Bank, Bahamas. (**c**) Color palette for bathymetry, depths are in centimeters.

Processing of two full LANDSAT-8 scenes at a 15 m pan-sharpened GSD is customarily done in just one day. This allows one to undertake time series study cases, in comfortable conditions, to produce a final clean combined depth SDB, free of transient adverse local conditions or areas of no data, which does not need any smoothing, at a 15 m GSD: See Figure A1a in Appendix B. This is presented in another paper [14].

### 4.2. Methodology

↘ For detail on the 4SM methodology, please refer to Appendix A, and also to [26].
↘ **The practitioner displays the image, and creates some shapefiles:**
  ✦ For an import window and for various ROIs (bad data, glint, deep water, bare soils, dense vegetation).
↘ **Then the practitioner runs the 4SM AutoCalibrator routine:**
  ✦ This creates the working database,
  ✦ then imports (and resample as required) raw image data,
  ✦ then samples the image for deep water radiance, glint, bare soils, and dense vegetation,
  ✦ then produces glint regressions,
  ✦ then evaluates the Soil Line,
  ✦ then produces a mask,
  ✦ then extracts the calibration data from the image (BPL pixels, bi-dimensional histograms) for all pairs of bands,
  ✦ then formats and displays the image-specific auto-calibration command line script,
  ✦ then computes and displays an auto-calibration diagram,
  ✦ then stops.
↘ **Then the practitioner runs multiple iterations of the 4SM Calibrator routine in order to fine-tune the following parameters in the command line script:**
  ✦ *K*_blue_/*K*_green_, the ratio of attenuation coefficients;
  ✦ spectral *LsM*, the maximum brightness of shallow bottoms;
  ✦ spectral *La*, the atmospheric path radiance, and therefore spectral *Lw*, the water volume reflectance;
  ✦ spectral *Lm*, radiance thresholds.
↘ **Once satisfied with the optical calibration for all pairs of bands, the command line script is entirely specified for the current scene. Then the practitioner runs the 4SM Modeler routine:**
  ✦ For each line, raw 16 bits spectral data are read, scaled to the 8 bits range, de-glinted, pan-sharpened, and smoothed if required;
  ✦ then the inverted RTE is applied on the fly to each shallow pixel.
↘ **This yields**
  ✦ retrieved depth *Z* in units of meters,
  ✦ spectral water column corrected reflectance in relative units of radiance,
    - which may then be converted to calibrated reflectance,
    - and classified if bottom type signatures are available.
↘ **Various tools are available** for recoding, profiling, sea truth regression, depth residuals, bottom typing, and combining depths.

**Figure 3:**
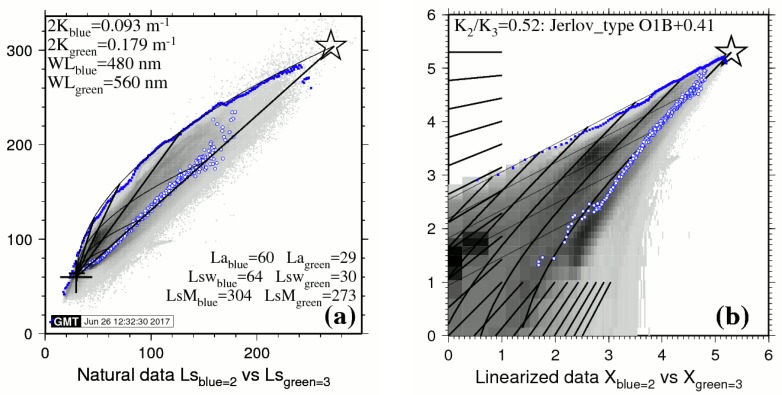
(**a**) Calibration diagram for natural data. (**b**) Calibration diagram for linearized data.

Figure 3a,b present the optical calibration diagrams for the pair Blue = 2 versus Green = 3 of the LANDSAT-8, 29 January 2014, image over Lee Stocking Island, Bahamas (Tongue of the Ocean).

The backdrop of bi-dimensional histogram in shades of gray represents all image pixels; all marine pixels are de-glinted. Blue dots: The BPL pixels are real pixels, each referenced to its row/line position in the image. Small empty blue circles represent the scatter of averaged bare land pixels, as a proxy for the Soil Line. The values of select parameters are provided. Thick lines: Six isobath lines are drawn: 0, 5, 10, 15, 20 and 25 m. Thin lines: Five isobottom lines are drawn.

Band 2 is Blue; band 3 is Green. The ratio *K*_blue_/*K*_green_ = 0.52. It is observed that the scatter of BPL pixels in the plot of linearized data of Figure 3b forms a fairly straight line with a slope *K*_blue_/*K*_green_ ≈ 0.52 over the whole 0–30 m depth range for bright bottom substrates: This slope represents the clearest waters over the scene. This value of *K*_blue_/*K*_green_ forms the basis of the so-called “self-calibration”: Using this value, spectral K for all visible bands are then interpolated in units of m^−1^ using Jerlov’s data: This yields *K*_1_ = 0.099; *K*_2_ = 0.093; *K*_3_ = 0.179 and *K*_4_ = 0.675. Still, the plot of natural data exhibits distinct bumps: These bumps indicate that things might actually be not so simple after all; and it must be noted that this process may hide areas of less clear waters for which the ratio *K*_blue_/*K*_green_ exceeds 0.52. It is also clear in Figure 3b that the maximum optical path 2*K*·*Z* (i.e., the maximum depth of bottom detection) decreases drastically as the bottom brightness decreases.

## 5. Results: Some Sea Truth on Depths Retrieved at Caicos Bank

In the end, all parameters that are necessary to specify the simplified RTE for visible bands are available, and the image may be processed. The RTE is inverted using Equation (3) and the PAN solution (see Section 2.2), without any smoothing. We shall now use a DTM over Caicos Bank to investigate the following two questions: (i) is it safe to use the Jerlov assumption to specify spectral 2*K*? (ii) does the depth residuals ZDTM-ZSDB represent noise?

An existing sea truth DTM raster of Caicos Bank, Bahamas was purchased over the internet, see Figure 2b; it is used to evaluate the SDB obtained using LANDSAT-8 image dated 8 November 2014 at 30 m GSD, over the 0–30 m depth range. This image subset is ~120 km wide, 4018 rows and 4149 lines: That is 16.7 million pixels. Shallow bottoms at Caicos Bank are commonly covered in turtle grass; they also exhibit areas of very bright oolith sands. As regards the accuracy of this DTM, following are relevant excerpts from [24]: “*An excellent 1:200,000 scale navigation chart, Turks & Caicos Islands, was scanned... Segments of some of the contours were digitized to add control to the DTM... Almost 3700 soundings were collected and converted to points, each with an x, y location and water depth. … Additional control was provided by soundings collected in the NW portion of the platform. … To fill in gaps, water depths were interpreted from the Landsat imagery. For example, along the reefs soundings were not available, so an arbitrary water depth of 0.2 m was assigned where surf was seen on the imagery. … The ~4000 control points and 230 control lines were modeled using a triangulated irregular network (TIN), then converted to a 30-m grid (UTM Zone 18N, WGS-84).*” Strong zooming on Figure 2b reveals details of the location of depth sounding data. In summary, a detailed evaluation of the accuracy of the proposed method would require a much better quality DTM; but this coarse DTM should adequately serve the main purpose of this evaluation, which is primarily to test the assertion that there is no need for field data to calibrate the simplified RTE.

**Figure 4:**
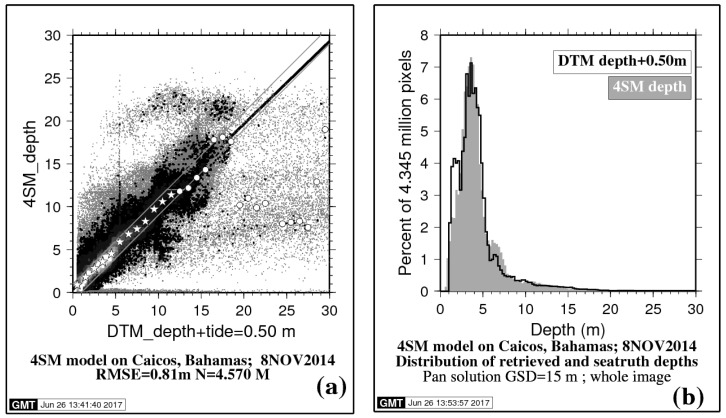
(**a**) Sea truth regression of SDB versus DTM depths for the Caicos Bank study case: Plot of 4SM depth versus DTM depth, allowing for a 0.5 m tide height. Outliers: Thin gray points are excluded from the regression. (**b**) Sea truth regression of SDB versus DTM depths for the Caicos Bank study case: Overlay of histograms of retrieved depths and sea truth depths; both histograms account for all depth points used in the regression.

In respect of this study, this DTM is found wanting:

- Depths appear to be overestimated in the 1–2.5 m depth range, as can be seen in the sea truth histogram of Figure 4b; the 3–6 m range is prominent; the 15–30 m depth range is poorly represented as the bottom drops suddenly to the deep;
- several areas lack depth points in sensitive surroundings such as clusters of isolated shoals, and in the 15–30 m range;
- no water depth datum is available for this DTM, and tide height is unknown, although it should not exceed 0.8 m. As it turns out, a tide height of 0.5 m appears to suit the situation.

Nevertheless, this DTM shall be very useful, subject to the some manipulations, which are necessary for the Root-Mean-Square Error (RMSE) on SDB depths to mean anything.

### 5.1. Some Preparation

Because of the unusual nature of the DTM, a fairly complicated protocol was implemented, which involves sub-sampling and masking, then thresholding of outliers. The DTM was resampled to 15 GSD, in order to overlay on the Landsat pan-sharpened image.

Then a mask is used to exclude areas where (i) the sea truth DTM raster exhibits coarse triangular interpolation patterns; (ii) the LANDSAT-8 image exhibits scatters of very small clouds/shadows. For this exercise, and because the project area is so vast at 15 m GSD, one in three lines and one in three columns are read; that is one in nine pixels. Pixels that do not exhibit both DTM depth and SDB depth are also excluded. This results in a total of 581,982 pixels (or depth pairs) to be evaluated, which therefore represent 5,237,838 raster nodes (or depth pairs).

Then the 5,237,838 accepted depth pairs (in units of centimeters) are assigned to decimeter bins. Some bins count many pairs (like in the 30 to 60 decimeter depth range); they form the main body of data to be regressed and shall be plotted from black to white as thick points in the bi-dimensional regression of Figure 4a. However, other bins count very few pairs. These are outliers and shall be plotted as thin gray points. Outliers occur for many different reasons and must be excluded from the regression. For example, bad areas in the DTM (coarse spacing of depth sounding), or in the image (clouds and their shadows), are flagged using a mask and excluded from the regression. Moreover, depth bins that count too few pixels are also flagged using a threshold and excluded: In practice, a threshold of two pixels is enforced, i.e., pairs with fewer than three occurrences are flagged and excluded.

### 5.2. Sea Truth Regression

Finally, all accepted pairs are binned into meters and averaged: They are represented by stars and circles in Figure 4a: From 0 to 12 m, these averages display well along the diagonal (stars). In contrast, deeper than 12 m, other averages are mostly outliers, which must be discarded (circles). This 12 m threshold depth is determined as follows: Starting from 0 m, all accepted pairs are counted by order of increasing DTM depth until a total of 99% of all accepted pairs is reached. This yielded 12 m. In other words, DTM depths in excess of 12 m only account for 1% of all accepted pairs for this scene (circles). By application of this threshold, the regression now is faithfully representative of the 4SM approach over the 0–12 m depth range for one single LANDSAT-8 image in the Bahamas: Allowing for a tide correction of 0.5 m on DTM depths, the following regression is obtained:

(13) SDBdepth=0.35+0.96×DTMdepth,N=4.6million,R2=0.89,RMSE=0.81m.

Inside the gray polygon of Figure 4a, SDB depths that are within ±1 m of DTM depth amount to 89.6% of all black to white points.

### 5.3. Learning from the RED Solution: Using a LUT

One must question the cause of the wide scatter of points off the diagonal in Figure 4a. In the 4SM approach, inverting the RTE relies on the Soil Line assumption, which can only yield underestimated retrieved depths over green shallow substrates (which cover most shallow bottoms at Caicos Bank) when using either the Green solution or, to a lesser extent, the Pan solution. In order to alleviate this problem, one may first learn from the results obtained by using RED solution a way to adjust the Green and the Pan solutions in order to improve the results for a majority of pixels, and write this knowledge into a look-up table that is hard-coded in the 4SM code, then use this LUT in place of the original Soil Line. This device turns out to be quite beneficial in producing a less biased scatter of points in the bi-dimensional regression histogram. These depth residuals affect areas in the image rather than pixels at random (see an illustration in Section 5.5). Therefore, they are not noise-related, but rather represent spatial variations in the spectral properties of the bottom substrate or of the optical properties of the water, which are not accounted for by the inversion scheme. This is inevitable, and should be linked to variations in the local conditions of the bottom substrate through space and/or time, such as the life and death of biofilms, the seasonal vegetation cycle, etc., and, in the case of the Bahaman karstic shelf, bursts of discolored ground water seeping up from the shallow brackish water table.

### 5.4. No Need for Field Data

Applying a smart smoothing scheme upon running the inversion of the RTE does result in lesser scatter of points in the bi-dimensional regression histogram. Restricting the depth range to 3–17 m should also reduce the RMSE on retrieved depths, because the 0–3 m depth range is overrepresented in the DTM, as shown by the sea truth depth histogram of Figure 4b.

In the end, Figure 4a shows that, allowing for a 0.5 m tide height, depths retrieved in the 1–18 m depth range by use of the spectral 2*K* derived from the image itself are very close to the sea truth data. This is demonstrated by the quasi-diagonal scatter of 89.6% of all depth pairs, where retrieved depths are within ±1 m of sea truth depth; it clearly means that the optical calibration is correct, although no field depth data were used to specify it.

This shall also be verified by Favoretto et al. [14]. So far, it proved to apply to clear waters for all airborne/spaceborne hyper/multi-spectral imageries. Therefore, in a large majority of study cases over clear waters, it has been verified that, using the 4SM approach, there is no need for field data, nor for formal atmospheric correction, to calibrate the RTE and compute depths in meters: these SDB depths then only need a tide correction, even though they entail possibly severe depth residuals.

### 5.5. Accuracy of SDB Depth: Depth Residuals DTMdepth-SDBdepth

An evaluation of the accuracy of the method for retrieved depths by UKHO standards [27] would require a much smaller GSD and a LIDAR or MBES dataset. In Favoretto et al. [14], the SDB depths using LANDSAT-8 images shall be compared to genuine depth sounding data.

**Figure 5:**
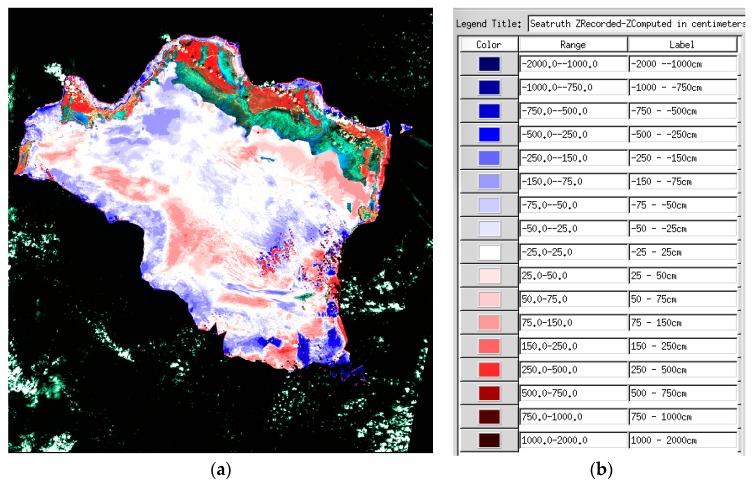
(**a**) Depth residuals ZDTM-ZSDB. Display of the overlay of the depth residuals ZDTM-ZSDB over a false color composite. Please beware that green areas represent the backdrop of false color composite over extremely shallow areas, while deep red areas represent dense vegetation on land. (**b**) Color pallet for depth residuals ZDTM-ZSDB.

For the present study, we only have 30 m GSD images and a coarse DTM. SDB depths are computed in centimeters; by subtracting the SDB depths from the tide-corrected DTM depths, one obtains a raster of the depth residuals for LANDSAT-8 image dated 8 November 2014. This is presented in Figure 5a. Changes in color are by steps of 0.25 m (0–1 m, light hues), then by steps of 1.0 m (darker hues). Residuals less than ±0.25 m are mapped in white. Blue hues represent areas of overestimated depth. Red hues represent areas of under-estimated depths.

Some areas are badly affected by clutters of small clouds and their shadows, and some odd dark blue areas are affected by erratic interpolation in the DTM. Still, most depth residuals affect areas rather than individual pixels, and therefore signal areas where water column correction was too weak or too strong, rather than noise.

It may be seen all along the northern edge reef rim at Caicos Bank, that many dark blue areas exhibit badly overestimated depths while some dark red areas exhibit badly underestimated depths. These reef rim locations depart from the vast bank, which is covered in turtle grass (see Section 5.3). Much more need to be investigated in order to try and alleviate these discrepancies, as the Soil Line assumption is certainly not a match for a spectral library of bottom substrate spectral reflectance signatures representative of this vast site.

## 6. Discussion

Remote sensing of the ocean color over an optically shallow bottom involves equations that describe the fate of a photon from the sun to the water surface, then down through the water column to the shallow bottom, then up through the water column, then up to the remote sensor’s very narrow field of view. This is the domain of analytical methods and very advanced sensors. Analytical methods rely on a database of spectral bottom substrate reflectance, possibly obtained in situ, and aim at producing an exact solution for depth, spectral *K*, and water column corrected spectral bottom reflectance [16].

For sake of operationality, empirical methods have been devised in order to make use of available and affordable imagery, which have all been specified for land areas, like LANDSAT. Therefore, empirical methods overlook much of the analytical complexities. To this date, they all rely on existing in situ depth soundings to retrieve the shallow bottom depth, and do not produce the most desired water column corrected spectral reflectance of the bottom substrates, which would allow for bottom typing. After all, one must keep in mind that SDB can be useful even though the retrieved depths are biased to some extent, and that bottom typing depends on the separability of water column corrected bottom spectral signatures much more than on exact signatures. Therefore, the relevant question here is: What are the main advantages of a 4SM simplified RTE approach?

It must be stressed that this discussion assumes clear waters, with a negligible water leaving reflectance over optically deep waters in the RED-NIR-SWIR range of the solar spectrum, although significant progress in underway to accommodate less favorable water quality conditions [28,29].

### 6.1. De-Glinting, Deep Water Reflectance Lsw, and the Bottom Contrast

First, it must be acknowledged that some images exhibit such atmospheric or sea-surface complexities that they must be discarded altogether. Otherwise, in the absence of distinct wind-generated glint clutter at the water surface, glint regressions are prepared using lumps of haze or even atmospheric adjacency effect over deep water. A good de-glinting using the Hedley et al. protocol [30], which is based on the dark pixel assumption in the NIR band, produces an even radiometric spectrum over deep water areas: This is the condition for estimating the all-important radiance terms *Lsw* and *Ls* in the RTE (Equation (5)).

This de-glinting protocol proved to efficiently remove the swell modulated and adjacency effect components of the marine signal, but not the sky dome component, which is constant, i.e., not modulated by the swell or water surface glitter. This is not a problem, though, as the sky dome component affects both *Ls* and *Lsw* terms. Therefore the bottom contrast *Ls*-*Lsw* is not affected. As a result, and in most cases, the simplified RTE approach does not require a formal atmospheric correction.

The optical depth limit for inferring bathymetry, also known as the extinction depth, is ~30 m in the sea truth case at Caicos Bank (Section 5), where the water type is estimated as oceanic OIB of Jerlov. Seven circles in Figure 4a show that the 12 m threshold actually extends to in excess of 17 m. The depth threshold used for the sea truth regression makes some sense, as the retrieved depth below the threshold is hardly affected by the uncertainty on *Lsw*_PAN_ (in the case of the PAN solution), unlike the deeper range above that threshold, which is most sensitive to a slight increase or decrease of this parameter. However, the 4SM practitioner can—and must—investigate the water column corrected spectral reflectance result. Indeed, if *Lsw*_PAN_ is a bit too high, depths in the 17–30 m range will tend to be overestimated—more so as the bottom contrast gets close to extinction—and the water column corrected reflectance shall be overestimated accordingly; and vice versa. This shows in the display of a color composite of the water column corrected reflectance, and the practitioner must exercise caution and act accordingly by slightly adjusting the *Lsw*_PAN_ term. This discussion is related to the concept of a cut-off depth highlighted by the United Kingdom Hydrographic Office [27].

### 6.2. Path Radiance La, and Water Volume Reflectance Lw

The path radiance *La* needs not to be impeccable: What is needed is that the Soil Line be given the right spectral properties, i.e., ratios *LB_i_*/*LB_j_*. Negative radiances due to an approximate “atmospheric correction” cannot be tolerated: This is a severe limit to the use of established atmospheric correction protocols that rely on atmospheric models.

In the 4SM approach, this is easily prevented, as the *Lw* term simply cannot be negative. Therefore, the water volume reflectance *Lw* is easily estimated as *Lw* = *Lsw* − *La*. However, the practitioner needs to keep in mind the basics of water volume reflectance, and of its variation over the visible range as a function of spectral diffuse optical properties of the water in situ: The water volume reflectance term *Lw* is negligible over the NIR-Red range, then increases smoothly through the Orange–Green range, then increases sharply over the Blue range.

The depth retrieved over dark bottoms depends to a large extent on a correct estimation of the water volume reflectance term *Lw*, as may be verified by inspection of Figure 1a. This is an exclusive bonus of the 4SM approach over empirical approaches.

### 6.3. Spectral K, Mid-Waveband, and the Far-Green to Red Region

Spectral *K* is wavelength dependent in Jerlov’s data. Each multispectral wide band has its own response curve; therefore, it is relevant to consider its effective- or operational-wavelength rather than its wavelength at mid-waveband. In view of all the simplifications of the RTE, this question might seem a third order technicality, but it is not for multispectral wide bands: For example, the Green wide band of LANDSAT-8 (530–590 nm) extends well over a steep increase in *K* between 550 and 600 nm. Therefore, it just happens that using the ratio *K*_blue_/*K*_green_ observed in the image to interpolate *K*_blue_ and *K*_green_ at mid-wavelength then yields the right spectral *K* in m^−1^ over the whole visible range for use when operating the RTE. The end result is that Coef*Z* ≈ 1 in the following:

(14) FinalSDBdepth=CoefZ·SDBdepth–TideHeight,

where Coef*Z* and *TideHeight* can only be estimated using sea truth data.

There is a long way to go from Jerlov’s “diffuse irradiance attenuation coefficient *Kd*“ to operational *K* in the simplified RTE. Quite significantly, Jerlov [21] also commented: “*It should be borne in mind that, in the surface region 0–10 m, the irradiance attenuation coefficient Kd for high solar elevation is close to the absorption coefficient a*.” After all, one may consider that the path of bottom-reflected photons that reach the sensor’s very narrow field of view is likely to have been very close to the vertical, both on its way down from the sea surface to the bottom, and on its way up from the bottom to the sea surface, with most other photons being scattered out of this near-vertical path. If this holds true, then one may consider that rewriting the simplified RTE for irradiances (Equation (1)) into a new version for radiances, or digital numbers for that matter (Equation (2)), now finds its justification. This aspect certainly warrants further academic investigation using a radiative transfer numerical model.

### 6.4. Homogeneous Waters

The assumption of homogeneous waters may be considered a lost cause in many cases, even in atolls. However, some variations in the optical properties can be accommodated in the 4SM approach, to a limited extent. This is presented in Favoretto et al. [14].

### 6.5. Pan-Sharpening and the Use of the Panchromatic Band

The panchromatic band may be used without pan-sharpening of the multispectral bands: For this it must be oversampled accordingly from 15 to 30 m GSD. However the panchromatic band may be used with pan-sharpening of the multispectral bands: For this various packages are available. We experimented using the Rstudio software package for pan-sharpening, although the co-registration of the PAN band with other bands can be a problem. However, for better control and for operational reasons, it is practical to include a pan-sharpening loop in the 4SM work flow. This is done as follows: After undersampling the multispectral bands from 30 to 15 m GSD (a mere duplication of pixels), de-glinting (and before smoothing if required), let *Ls_N_* be the radiance in band *N* at the center of a five pixels kernel: First a low-pass filter (LPF) is extracted as the average of panchromatic radiance in the kernel, then the following convolution is applied:

(15) $$Ls_{N}=Ls_{N}\cdot Ls_{\mathrm{PAN}}/LPF.$$

So, pixels that are brighter than their surrounding in the PAN band get a boost in the multispectral bands, and vice versa. This turns out to be very efficient and fast. Therefore, the SDB and water column corrected rasters may be produced at 15 m GSD using LANDSAT-8 images.

In practice, pan-sharpening is only applied to the Coastal, Blue and Green bands of LANDSAT-8; other multispectral bands are not involved in the Pan or Green solution.

There is another advantage of using the panchromatic band. All existing methods only use multispectral bands; this works as long as there is sufficient color separation. For example in clear oceanic waters, *K*_blue_ < *K*_green_ < *K*_red_ and the ratio *K*_blue_/*K*_green_ is much less than 1. However, in Oceanic III or Coastal water types, there is hardly any color separation and the ratio *K*_blue_/*K*_green_ gets very close to 1 or even worse. This precludes a reliable estimation of the model parameters in methods like Lyzenga’s or Stumpf et al.’s. The PAN solution offers a valuable alternative, as there is always enough color separation, even with coastal water types.

### 6.6. Which Inversion Solution?

As exposed in Section 2.2, several solutions can be operated for the inversion of the RTE. For example, a LANDSAT-8 pixel that exhibits significant bottom contrast *Ls*_red_-*Lsw*_red_ in the red band may be modeled using the Red solution, but the Green solution is also available. For areas modeled using the Red solution, the ratio (*LB*_1_ + *LB*_2_ + *LB*_3_)/3*LB*_4_ is to a large extent determined by the bottom contrast in the red band, and much less on the bottom contrast in other bands 1, 2 and 3. This becomes critical as the bottom contrast becomes nearly extinct, i.e., very close to its maximum optical depth *K*_red_·*Z*max_red_: *Lsw*_red_ may be just a little too high and cause overestimation of the retrieved depth, and vice versa. This kind of uncertainty hardly affects the neighboring pixel if modeled by the Green solution at just a fraction of its maximum optical depth *K*_green_·*Z*max_green_. As a result, an unsightly gap appears in the raster of retrieved depths along the frontier between areas modeled by either solution. Moreover, such gap tends to be either positive or negative and vary in intensity, as determined by slight local variations of the water’s optical properties. This becomes even more of a nuisance when more wavebands, and therefore more solutions, are available, like when using a hyperspectral image. The end-user is not prepared to tolerate such gaps in a SDB product, which cast doubts on the overall approach [31].

In this regard, apart from accommodating a pan-sharpened image and Coastal water types, a PAN solution offers the following distinct advantage: All shallow pixels are processed seamlessly using the PAN solution, without any gap, over the whole shallow depth range.

In practice, the Green bands are skipped out of the PAN solution, which uses the ratio Average*LB*_blue_/*LB*_PAN_, as this frees the Green water column corrected bands. In this regard, it does not make much difference whether the image is multispectral or hyperspectral, as the bottom contrast in the PAN band is what determines the retrieved depth.

### 6.7. Transient Heterogeneities and Combining Depths

Transient heterogeneities cause very adverse artifacts, which cannot be avoided. Favoretto et al. [14] show how a time series of LANDSAT-8 images is used to produce a combined depth raster that is free of transient encumbrances (clouds/shadows, seasonal variations, local blooms, heterogeneities of the atmosphere and water properties, boats and their wakes, etc.). This raster may then be considered as a 15 m GSD DTM over the scene, which is tested against a quality sea truth dataset. This is illustrated in Figure A1 of Appendix B.

### 6.8. Bottom Typing

Water column corrected spectral bottom reflectances may be converted to calibrated reflectances, in the range 0–1, by use of the metadata provided with the LANDSAT-8 or WORLDVIEW-2 imagery. However, because of the depth residual, they are only a proxy to real bottom substrate reflectances. Nevertheless, they should prove useful in the case of time series monitoring.

The PAN solution is also beneficial for the purpose of bottom typing: First it frees the water column correction of the green band when computing the ratio (*LB*_1_ + *LB*_2_)/2*LB*_PAN_; then all pixels are processed using the same solution over the whole depth range. This is a distinct bonus for bottom typing. This is illustrated in Figure A1 of Appendix B.

### 6.9. Applicable to Clear Marine and Fresh Waters Worldwide

It may easily be realized that the 4SM approach applies to all shallow waters, worldwide, as it does not use any existing information that is specific to the study case site. It was even applied on river sites, and on the ice shelf of Greenland.

### 6.10. The 4SM Approach Improves Markedly over Stumpf’s Log Ratio Method

**Figure A3:**
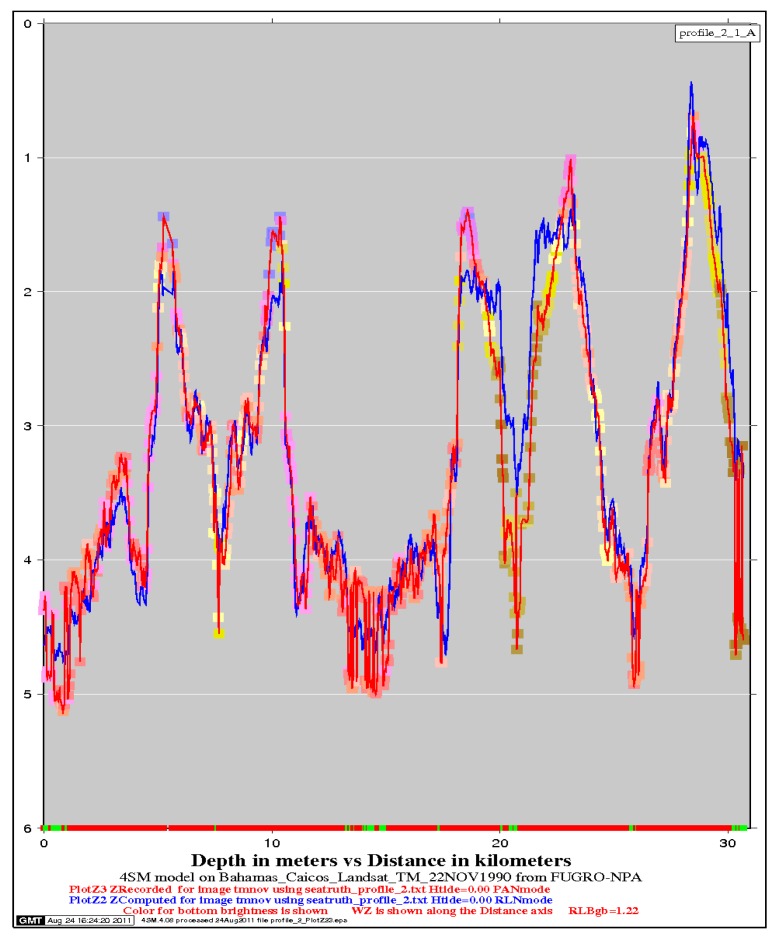
Computed depth versus distance.

Once 4SM depth is available, it can be used to calibrate the parameters of Stumpf’s log ratio method [2]. Results by these two methods are compared in Figure A3 of Appendix C. It clearly shows that depth is overestimated by the log ratio method over bright bottoms, and underestimated over dark bottoms.

### 6.11. Work Flow and Performance

In the past, we have done the calibration and processing of the 4SM approach, painstakingly using band math in ERDAS and a spreadsheet, for student training purposes. Then we set out to write the 4SM code to cover the whole operation “on the fly”, from (i) reading the raw 16-bits image; (ii) to de-glinting, and smart-smoothing or pan-sharpening; (iii) to extracting calibration data; (iv) to working out all parameters required to specify the simplified RTE for the scene under study; (v) then finally to processing the image, line by line, pixel-wise, within hours of downloading the raw data. Then on occasion (vi) we proceed to combining depths from a time series of images to produce a complete and clean DTM of the shallow areas.

## 7. Conclusions

The simplified RTE has been presented, with details on how it is operated in the novel 4SM algebraic model-based ratio approach to satellite derived bathymetry and water column correction, based on the experience gained over 23 years. Then we presented most operational aspects of the optical calibration of the simplified RTE for a multispectral image, using several new assumptions. And a coarse DTM was compared to the SDB over Caicos Bank, Bahamas, at 30 m GSD. Following are the main conclusions.

De-glinting removes sea surface clutter, haze, and even atmospheric adjacency effect; therefore, in most cases, the dark pixel subtraction in the 4SM approach achieves an acceptable atmospheric correction. By application of the basics of the optical properties of atmosphere and water, an acceptable control is secured on the water volume reflectance *Lw*, and therefore on the atmospheric path radiance term *La*. This potentially yields more acceptable results over dark bottoms.

Regarding the specification of spectral *K* in unit of m^−1^ for the image under study, it is demonstrated that it can be obtained using only the image and Jerlov’s data for clear marine waters, near nadir viewing, and sun high in the sky. Therefore, there is no need for field data to calibrate the simplified RTE for clear waters, worldwide, although this aspect certainly warrants further academic research.

Regarding the panchromatic band, pan-sharpening of the multispectral bands showed to be beneficial and practical. For a hyperspectral image, a synthetic panchromatic band can be produced, and used in addition to spectral bands. We have commented that, apart from a smaller GSD, the panchromatic solution brings about several distinct advantages, in particular in the adverse case of Coastal water types of Jerlov.

Finally, from reading the raw spectral data to writing out the depth and water column corrected reflectance rasters, everything is done using one single executable 4SM code on a LINUX portable workstation, under tight control of the practitioner using one single command line shell script.

## Figures and Tables

**Figure A2:**
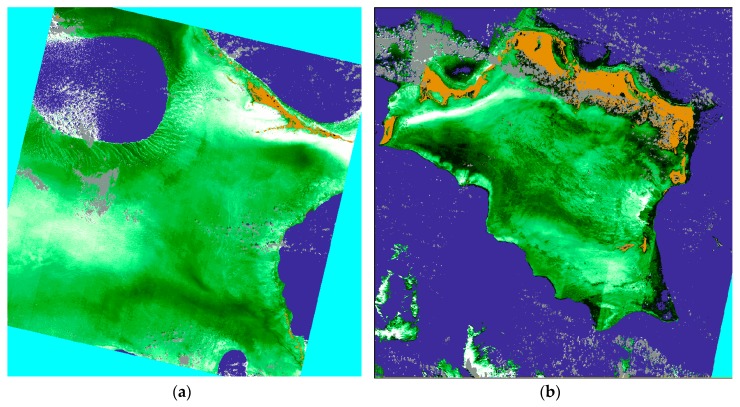
(**a**) 4 February 2016 over Lee Stocking Island; (**b**) 29 March 2014 over Caicos Bank.

## Appendix A. Detailed 4SM Work Flow: Refers to Section 4.2

### Appendix A.1. Image Preparation

All that follows is done using the 4SM code. First the raw data is imported as raw DNs into a working image database. If a panchromatic band is available, either it is oversampled and added to the multispectral bandset (e.g., for a 30 m GSD LANDSAT-8 seven bands study), or the multispectral bands are undersampled and the panchromatic band is added to the multispectral bandset (e.g., for a 15 m GSD LANDSAT-8 seven pan-sharpened bands study). In the case of a hyperspectral image, a synthetic panchromatic band is prepared as the convolution of radiance signal in each multispectral band *i* with the panchromatic response at wavelength WL*_i_*; then, it is scaled as appropriate and added to the set of multispectral bandset (e.g., for a 30 m GSD Hyperion study).

Once the raw data are imported in a working database, and for each processing run, the raw 16-bit data are accessed, scaled as floating points, de-glinted as required (some images do not require de-glinting, or cannot be de-glinted), smoothed if required, pan-sharpened if required, and finally processed as required on the fly, line by line, under control of the command line script.

Optical calibration requires the interactive preparation of a mask to delineate land from water. Shapefiles are prepared to mark out unwanted areas, alien floating objects, and regions of interest (ROI) where to sample the glint, the deep water reflectance, some brightest bare land, and some healthy vegetation.

### Appendix A.2. De-Glinting

De-glinting uses the process described by Hedley et al. [30], which assumes a null penetration in water of the NIR-SWIR bands of the spectral images. A sample of marine pixels over optically deep water areas affected by a variable amount of sun or sky glint, or by lumps of haze, or even by atmospheric adjacency effect, is collected in order to perform the linear regression of all visible bands against the NIR or SWIR band. Then the amount of “glint” in visible bands is evaluated and subtracted using these regressions.

### Appendix A.3. Extraction of the Calibration Data

The mask and various shapefiles are used to ensure that only the desired areas or ROIs are sampled for calibration data: “Clean” shallow marine pixels are wanted. This extraction is done for whole image, or through a mask, or limited to a sub-window. To extract the BPL pixels for the pair of bands *i* and *j* with *K**_i_* < *K**_j_*, for each *Ls**_j_* the pixel with the highest *Ls**_i_* is selected along with its position in the image. This forms the BPL pixels for that pair of bands. It is at times useful to enable smoothing. Bi-dimensional histograms (BDH) are extracted for all pairs of bands *i* and *j* with *K**_i_* < *K**_j_*. Shallow marine pixels can be raw or de-glinted. The BDH can be complete or marine areas only, or even land only. In the end two calibration data text files are written, one for the BPL data, and one for the BDH data.

Upon the first operation, a provisional “auto-calibration” command line script is formatted [32]: It offers default or rough estimate values for the desired parameters; the auto-calibration graph is displayed automatically on screen.

The calibration data for the specific image to be processed are now available as text files: Appendix A.4 provides details of how they are used to work out the complete set of spectral parameters that are necessary to specify the simplified RTE for visible bands. This usually takes less than one hour for a LANDSAT-8 image.

### Appendix A.4. Optical Calibration for the Multispectral Bands

Then these preliminary values must be edited and modified as appropriate in that script by the practitioner, and a new calibration run is launched automatically upon closing the screen display of the previous calibration run, until satisfied. These parameters form the backbone of the command line script. This step requires all the attention of the practitioner.

- Ratio *K*_blue_/*K*_green_: Must be adjusted manually.
- *LsM*: Must be adjusted manually together with the ratio *K*_blue_/*K*_green_, so as to ensure a nice fit of the BPL model with the scatter of BPL pixels.
- As for visible bands other than Blue and Green, their *K* and *LsM* values must be adjusted manually, so as to ensure a nice fit of the BPL model with the scatter of BPL pixels.
- *Lsw*: Choosing the location where to estimate the deep water reflectance is of the utmost importance. De-glinting is essential; spectral profiling across the image may help refine this choice.
- *Lw*: Because *La* = *Lsw* − *Lw*, the choice of *Lw*, along with that for *LsM*, completes the specification of the Soil Line. For this, one conveniently remembers that:
  ↘ *Lw* may be considered negligible over the red range, as it is for a NIR or SWIR band;
  ↘ dense healthy vegetation pixels should plot:
    ✦ near the Soil Line for a Blue/Red pair of bands because both Blue and Red are darker than Green, but
    ✦ on the green side of the Soil Line for a Green/Red pair of bands.

### Appendix A.5. Optical Calibration for the Panchromatic Band

A panchromatic band is specified by its response curve, which covers a specific portion of the visible-NIR range. Therefore, its diffuse attenuation coefficient *K*_PAN_ may be expected to decrease more or less regularly as the water depth *Z* increases. This translates graphically into a curved linearized BPL model. This curve is specified as a function (i) of the panchromatic response curve; and (ii) of the value of *K*_blue_ over the whole depth range.

### Appendix A.6. Iterations and Fine Tuning

The optical calibration of the simplified RTE is now secured. However, it is often the case that some local conditions depart notably from the overall picture. It is important that the practitioner investigates such odd situations in order to refine the choice of spectral deep water radiance, or the spectral threshold to be applied to radiances. This may be done by displaying the spatial variations of spectral raw and/or de-glinted radiance using a profiling routine available in the 4SM code. This is very much part of the calibration process and should not be overlooked for the sake of expediency.

## Appendix B. Some SDB and Bottom Typing Maps: Refers to Section 6.7 and Section 6.8

Figure A2 presents the spectral angle mapping of bottom type: The water column corrected bottom signature is converted to reflectance, then coded into the spectral angle for Blue-Green bands, using Landsat-8 images. Spectral angle mapping is insensitive to bottom brightness.

## Appendix C. Comparison with Stumpf’s Log Ratio Method: Refers to Section 6.10

Figure A3 displays computed depth along a 30 km long profile at Caicos Bank, using a Landsat TM image. The Blue profile represents depth computed by 4SM using Stumpf’s log ratio method [2]. The Red profile represents depth computed using the 4SM method. Colored squares represent bottom brightness. Bright bottoms are colored purple-blue, while dark bottoms are colored brown.

## Appendix D. Computer Software

A basic version of the 4SM executable code is available, along with a tutorial using a subset of a SPOT 1 image of Tarawa atoll, Kiribati. It may be accessed at [33]. A demonstration is available for anyone to run on their own LINUX workstation, complete with 4SM executable code, image data and command line scripts; it uses a 17 bands CASI image of Heron Island, Great Barrier Reef, Australia. The output is rasters of de-glinted bands, SDB, rasters of water column corrected bands, bottom typing, and sea truth validation. This demonstration is available at [34].
